# Carriage of HLA-DRB1*11 and 1*12 alleles and risk factors in patients with breast cancer in Burkina Faso

**DOI:** 10.1515/biol-2021-0113

**Published:** 2021-10-11

**Authors:** Abdou Azaque Zouré, Lanyo Jospin Amegnona, Nayi Zongo, Isabelle Touwendpoulimdé Kiendrebeogo, Pegdwendé Abel Sorgho, Fabienne Ingrid Zongo, Albert Théophane Yonli, Herman Karim Sombié, Aboubacar Hierrhum Bambara, Alexis Yobi Sawadogo, Marie N. L. Ouedraogo, Lassina Traoré, Sidnooma Véronique Zongo, Doriane Tatiana Lallogo, Bapio Valery Jean Télesphore Elvira Bazié, Théodora M. Zohoncon, Florencia W. Dijgma, Jacques Simpore

**Affiliations:** Departement of Biochemistry and Microbiology, Laboratory of Molecular Biology and Genetics (LABIOGENE), UFR/SVT, University Joseph KI-ZERBO, 03 P.O. Box 7021, Ouagadougou 03, Burkina Faso; Departement of Molecular Biology, Pietro Annigoni Biomolecular Research Center (CERBA), 01 P.O. Box 364, Ouagadougou 01, Burkina Faso; Department of Biomedical and Public Health/Institute of Health Sciences Research, (IRSS/CNRST), Institute of Health Sciences Research, 03 BP 7192, Ouagadougou 03, Burkina Faso; Department of Surgery and Surgical Specialties, Service of General and Digestive Surgery, University Hospital Centre-Yalgado OUEDRAOGO, UFR/SDS, University Joseph KI-ZERBO, 03 BP 7021, Ouagadougou 03, Burkina Faso; Department of Medicine and Medical Specialties, Service of Oncology, University Hospital Centre-BOGODOGO, University Joseph KI-ZERBO, UFR/SDS, 03 BP 7021, Ouagadougou 03, Burkina Faso; Department of Gynecology-Obstetrics, Service of Gynecology, University Hospital Centre-BOGODOGO, University Joseph KI ZERBO, UFR/SDS, 03 BP 7021, Ouagadougou 03, Burkina Faso; Faculty of Medicine, University Saint Thomas d’Aquin, 06 BP 10212, Ouagadougou 01, Burkina Faso

**Keywords:** breast cancer, HLA-DRB1*11, HLA-DRB1*12, risk factors, Burkina Faso

## Abstract

Several factors contribute to the development of breast cancer, including the immune system. This study is aimed to characterize the carriage of human leukocyte antigen (HLA)-DRB1*11 and 1*12 alleles in patients with breast cancer. This case-control study consisted of 96 histologically diagnosed breast cancer cases and 102 controls (cases without breast abnormalities). A multiplex polymerase chain reaction (PCR) was used to characterize the carriage of HLA-DRB1*11 and 1*12 alleles. The HLA-DRB1*11 allele was present in 26.59% of cases and 22.55% of controls. The HLA-DRB1*12 allele was present in 56.63% of cases and 55.88% of controls. This study found no direct association between the carriage of the HLA-DRB1*11 and HLA-DRB1*12 alleles and the occurrence of breast cancer. In addition, the deletion of the HLA-DRB1*11 allele is associated (beneficial effect) with obesity/overweight (OR = 0.13; 95% CI [0.01–1.14]; and *p =* 0.03) which is a risk for breast cancer. No direct association was found between the carriage of HLA-DRB1*11 and 1*12 alleles and breast cancer risk. However, further investigation of other HLA alleles involved in the occurrence of breast cancer may provide more information.

## 1 Introduction

In 2020, according to the GLOBOCAN 2020 database, cancer was diagnosed in over 19.3 million people, causing 10 million deaths. At the same time, female breast cancer was ranked the most diagnosed type of cancer in the world. In fact, 2.3 million new cancer cases were recorded, representing 11.7% of all diagnosed cancers, with 68,496 deaths, representing 6.9% of cancer deaths [1]. Africa is reporting an increase in breast cancer cases [1]. In Burkina Faso, breast cancer ranks first in terms of prevalence, i.e., 30.73 patients per 100,000 inhabitants, and second in terms of mortality, i.e., 20.3% of deaths due to cancer [1]. The high mortality from breast cancer in Africa is related mainly to very late diagnosis, whereas, in developed countries, better treatments are achieved based on early diagnosis. Globally, in the types of breast cancer, there are inherited (5–10%), familial (15–20%), and sporadic (75%) forms [2,3]. A combination of mutations in specific genes would confer risk and are classified as high, moderate, and low risk [4]. Thanks to molecular markers and hormone receptors, various subtypes of breast cancer responding to different treatments can be identified [5,6]. Several mutations on several genes are evoked, of which the mutations on the genes BRCA1, BRCA2, and TP53, which are involved in the repair of the DNA, are of high penetrance with a low frequency. Whereas the CHECK2, ATM, BRIP1, and PALB2, which are also involved in repairing the DNA, are of moderate penetrance [2,4,7,8]. Also, mutations in CASP8/10 (involved in apoptosis), β1-TGF, FGFR2, MAP3K1, LSP1 (involved in cell growth/cell signaling), and TNRC9 (probably encoding a transcription factor) are of low penetrance and frequency. Mutations in tumor suppressor genes such as PTEN, STK11, CDH1, NF1, NBS1, RAD50, and RAD51C are also associated with breast cancer risk [4,9,10]. In addition, the role played by the immune system in breast cancer in relation to the expression of human leukocyte antigen (HLA) genes involved in the anti-tumor immune response is being increasingly studied. The major histocompatibility complex (MHC) carries approximately 220 genes encoding proteins, more than half of which are directly involved in immunity, including the HLA gene system. The HLA genes are subdivided into three regions classified as HLA I, HLA II, and HLA III. Class I includes HLA-A, HLA-B, and HLA-C, while class II includes HLA-D with the subtypes HLA-DO, HLA-DP, HLA-DQ, and HLA-DR [11].

The HLA system has approximately 28,938 alleles, including 21,040 class I and 7,898 class II alleles “http://hla.alleles.org/nomenclature/stats.html” [12]. Allelic polymorphism of the HLA gene system is associated with various diseases. Therefore, other studies have focused on the role of the immune system in carcinogenesis by exploring the HLA. Indeed, the immune system plays a crucial role in tumor growth by eliminating, monitoring, and even promoting cancer cells [13]. Some genes of this system are involved in adaptive immunity via the MHC class II and appear to be significant players in the anti-cancer response through antigenic presentation and coordination of the immune response [14].

At the MHC class I level, two pathways of escape from the immune system by cancer cells are predominant: alteration in the expression of MHC class I [15]; and a complete or partial selection of phenotypes having lost the full expression of HLA I [16]. The alteration may include a total loss of HLA expression, loss of HLA haplotype, downregulation of specific HLA loci (HLA A, HLA B, and HLA C), allelic loss of HLA, and a combination of all these phenotypes [17]. The loss of specific HLA I expression leads to a deficiency in the presentation of immunodominant antigens and thus, allows an escape from the action of cytolytic T lymphocytes (CTC) but exposes to a “Natural Killer” (NK) response [18]. For example, in breast cancer, HLA I is not expressed in 96% of cases. The loss of specific haplotypes of HLA I allows for both CTC and NK to escape and promotes tumor progression. Expression of HLA A and B variants that bind with high affinity to NK inhibitory receptors leads to cancer cell escape from NK action. Overexpression of HLA E, associated with underexpression of HLA A, HLA B, and HLA C would play a role in NK evasion.

Moreover, HLA G is involved in immune evasion during breast carcinogenesis [19,20]. Furthermore, like lymphocytes and NK, immune cells can also be the site of certain dysfunctions in malignant breast tumors [23]. Other mechanisms such as downregulation of tumor-associated antigens, alteration of the apoptosis program, and the expression of inhibitory cytokines or disregard of the immune response have been reported [17,18,22,23]. Also, the modulation of the metastatic phenotype is associated with the modulation of the expression of MHC class I genes [24].

As for MHC class II, the escape of cancer cells from CD4+ T cells seems to be related to the normal functioning of the immune system. Indeed, two facts explain the inefficiency of T-helpers in tumor control: first, solid tumors do not express MHC class II, and therefore cannot directly stimulate CD4+ T cells, and second, tumor cells must be degraded by professional antigen-presenting cells (APCs) to be presented to T cells for action. However, cancer cells express MHC I of self and are therefore considered as self cells; this prevents any phagocytic action of APCs. Thus, the presentation of immunogenic tumor antigens is limited in quality and quantity in the tumor debris and secretion products that APCs can capture and present to TCD4+ [14].

Various studies have investigated a possible association of genes of the HLA system. HLA class I genes are by far the most investigated. As for HLA class II, HLA-DRB, and HLA-DQ are the most studied genes. In various populations, two alleles of the HLA gene, namely HLA-DRB1*11 and HLA-DRB1*12, have been associated either with protection against breast carcinogenesis or with a risk of breast cancer [25].

To our knowledge, no study has been conducted on the role of these two alleles in the occurrence of breast cancer in a sub-Saharan African population, particularly in Burkina Faso. Thus, our study aims to explore the carriage of HLA-DRB1*11 and HLA-DRB1*12 alleles in a Burkinabe population with unselected breast cancer (sporadic or family history/hereditary cases) to determine possible associations between HLA-DRB1*11 and HLA-DRB1*12 alleles and the susceptibility to breast cancer occurrence. This could contribute to the knowledge of breast cancer risk factors and a better prevention strategy of breast cancer in Burkina Faso.

## 2 Materials and methods

### 2.1 Materials

The study was conducted between October 2019 and March 2020. The study population (Burkinabe) consisted of 96 patients, histologically diagnosed with breast cancer (cases) and 102 without breast cancer (controls). They came for consultation to the University Hospital Centers: Yalgado OUEDRAOGO (CHU-YO) and BOGODOGO (CHU-B); at the Medical Centers with the surgical branch (Paul VI and Schiphra) in the city of Ouagadougou. Biomolecular analyses were performed at the Laboratory of Molecular Biology and Genetics (LABIOGENE) and at Pietro Annigoni Biomolecular Research Center (CERBA).

Women with breast cancer histologically confirmed by a pathologist were included in this study as cases, and women who came to the gynecological consultation for a pathology other than breast cancer had breast ultrasound scans, and were declared healthy concerning breast cancer were included as controls.


**Informed consent:** Informed consent has been obtained from all individuals included in this study.
**Ethical approval:** The research related to human use has been complied with all the relevant national regulations, institutional policies, and in accordance with the tenets of the Helsinki Declaration, and has been approved by The CERBA/LABIOGENE Ethics Committee.

### 2.2 Sample collection

After obtaining consent from patients and controls, a questionnaire was administered to collect sociodemographic, anthropometric, and clinical data from participants. Venous blood from consenting women was collected on Ethylene-Diamine-Tetra-Acetic (EDTA) filled tubes. After centrifugation, at 3,500 revolutions per minute for 15 min, the plasma and pellet were separated and stored at −20°C.

### 2.3 Multiplex polymerase chain reaction (PCR)

Genomic DNA was extracted from the blood pellet using the “Rapid Salting Out” technique modified and adapted from that described in 1988 by Miller et al. [26]. Using the BioDrop µLITE, DNA was quantified and purity was verified. Amplification of HLA-DRB1*11 and HLA-DRB1*12 alleles was performed using the primers described by Ma et al. [27], with a slightly adapted amplification program. This was a multiplex PCR targeting both alleles simultaneously, performed with the GeneAmp PCR System 9700 (Applied Biosystem, USA). The total reaction volume of 25 µL contains 9 µL of pure water (molecular biology grade), 10 µL of AmpliTaq Gold™ DNA Polymerase (10×, Applied Biosystems™), 0.5 µL of each of the three primer pairs [0.2 µM] (Table 1), and 3 µL of DNA [10 ng/µL]. The hepatocyte growth factor (HGF) primer pair is used for internal validation of the DNA amplification of each sample.

**Table 1:** Primers and amplicon size

Alleles	Primers	Size (bp)
HLA-DRB1*11	F 5′GTTTCTTGGAGTACTCTACGTC3′	176
	R 5′CTGGCTGTTCCAGTACTCCT3′	
HLA-DRB1*12	F 5′ACTCTACGGGTGAGTGTT3′	244
	R 5′ACTGTGAAGCTCTCCACAG3′	
HGF	F 5′CAGTGCCTTCCCAACCATTCCCTTA3′	432
	R 5′ATCCACTCACGGATTTCTGTTGTGTTTC3′	

The amplification program included an activation phase at 94°C for 10 min, followed by 35 cycles of denaturation series at 94°C for 60 s, hybridization at 56°C for 60 s, elongation at 72°C for 60 s, and finally extension at 72°C for 7 min. PCR products were run on a 2% of agarose gel. They were visualized under ultraviolet light at 132 nm using the GeneFlash revelation device (Syngene).

The sequences of the primer pairs used are given in the Table 1.

### 2.4 Statistical analysis

The study data were recorded into Excel before being processed with the Epi Info 7 software used for data analysis and interpretation. Frequency comparisons were performed with version 6 of the same software. Analyses were considered statistically significant at *p* ≤ 0.05, using the Fisher’s Exact test. Odds ratio (OR) and 95% confidence intervals (CI) were calculated to estimate associations between allele carriage and selected sociodemographic parameters of breast cancer.

## 3 Results

### 3.1 Sociodemographic characteristics

The mean age of the cases was 45.19 ± 0.90 years, and of the controls, 36.69 ± 1.06 years. About one-quarter (25.53%) of the patients were younger than 40 years. About 32.98% of these cases had been diagnosed with breast cancer before the age of 40. A risk of breast cancer was observed for the age group above 40 years (OR = 5.01; 95% CI [2.74–9.32]; and *p =* 0.001).

Body mass index (BMI) was calculated as the ratio of weight to height squared. These indices were then grouped into Normal/Lean (<25 kg/m^2^), Overweight (25–30 kg/m^2^), and Obese (≥30 kg/m^2^) according to the US National Institute of Health/National Heart Lung and Blood Institute (NCI/NHLBI) criteria. Patients and controls had a mean BMI of 29.77 ± 0.85 and 27.61 ± 0.76 kg/m^2^, respectively. These results found 34.91 and 24.51% obese in patients and controls, respectively. No association was found between overweight (OR = 1.4; 95% CI [0.59–3.64]; and *p =* 0.48) and obesity and risk of developing breast cancer (OR = 2.1; 95% CI [0.95–4.70]; and *p =* 0.07).

### 3.2 Family histories of breast cancer and other cancers

Patients in this study were asked whether their close relatives had confirmed breast cancer cases (10.63%) or other cancers (10.63%). No association was found between the patients’ relatives and the risk of developing breast cancer.

### 3.3 Medical treatment

**Figure 1:**
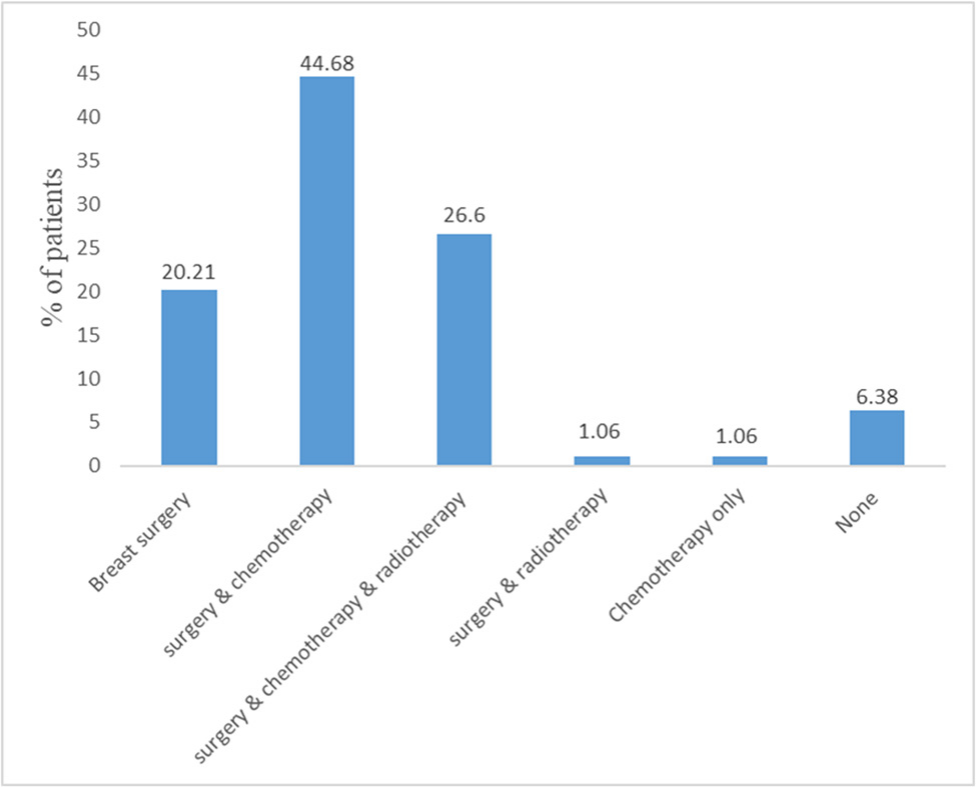
Treatments received by patients.

Three types of treatment (chemotherapy, radiotherapy, and surgery) were encountered in this population. Breast surgery was the most frequently performed treatment in 92.55% of all the patients. It was also performed in combination with either chemotherapy (44.68%) or radiotherapy (26.60%) (Figure 1).

### 3.4 Validation of PCR results

**Figure 2:**
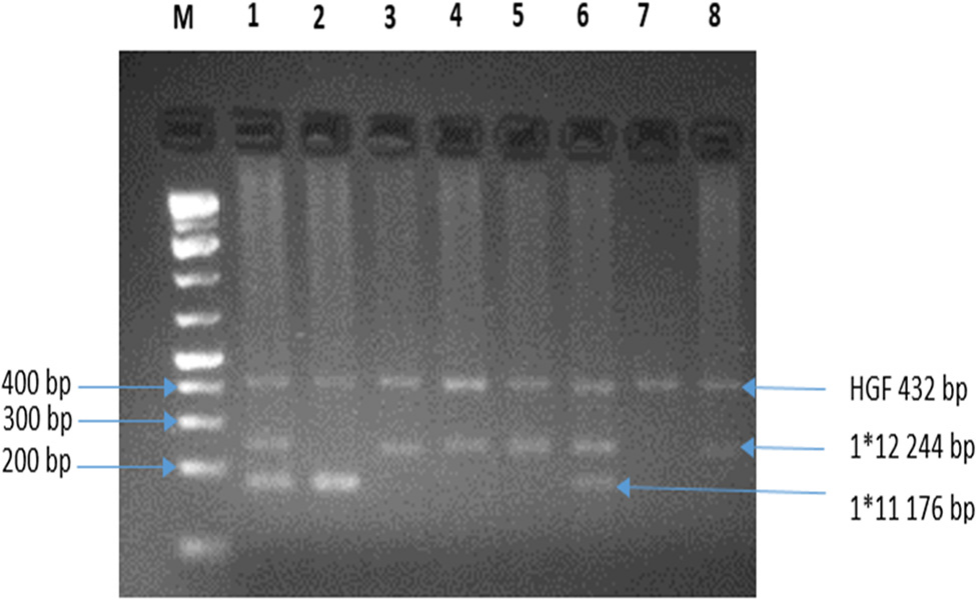
Electrophoresis gel of PCR products. Legend-M: Molecular weight marker. (1) and (6) Presence of HLA-DRB1*11 & HLA-DRB1*12; (2) Presence of HLA-DRB1*11; (3–5) and (8) HLA-DRB1*12, and (7) Valid PCR with the absence of the targeted alleles.

The validity of the PCR of a sample is based on the observation of an amplification band of the HGF gene which served as an internal control. The presence of HLA-DRB1*11 and HLA-DRB1*12 alleles are linked to a band at 176 bp and 244 bp, respectively (Figure 2).

### 3.5 Carrying of HLA-DRB1*11 and DRB1*12 alleles

The most common allele was DRB1*12, with a proportion of 56.63% in the general population of our study. The DRB1*11 allele was present in only 24.49% of the study participants. In addition, we did not find a relative risk of carrying both DRB1*11 and 1*12 alleles; this was true both for separate carriage and for combinations of carriage of these two alleles (Table 2).

**Table 2:** Carrying of the HLA alleles DRB1*11 and DRB1*12

HLA variable	*N* = 94	*N* = 102	OR (95% CI)	*p* value
	cases	controls		
DRB1*11				
Presence	25 (26.59%)	23 (22.55%)		Ref
Absence	69 (73.40%)	79 (77.45%)	0.80 (0.41–1.54)	0.61
DRB1*12				
Presence	54 (57.45%)	57 (55.88%)		Ref
Absence	40 (42.55%)	45 (44.12%)	0.93 (0.53–1.65)	0.88
DRB1*11 & 1*12				
DRB1*11+ & 1*12+	16 (17.02%)	13 (12.74%)		Ref
DRB1*11+ & 1*12−	9 (9.57%)	10 (39.8%)	0.73 (0.22–2.33)	0.76
DRB1*11− & 1*12+	38 (40.42%)	44 (43.14%)	0.70 (0.29–1.64)	0.51
DRB 1*11− & 1*12−	31 (32.98%)	35 (34.31%)	0.71 (0.29–1.72)	0.50

### 3.6 Association between some clinico-pathological parameters and the carriage of HLA-DRB1*11 and DRB1*12 alleles

No risk was found between both family history of breast cancer (Table 3) and carriage of the HLA-DRB1*11 and DRB1*12 alleles between cases and controls in this study. However, deletion of the HLA-DRB1*11 allele is associated (beneficial effect) with obesity/overweight (OR = 0.13; 95% CI [0.01–1.14]; and *p* = 0.03) (Table 4).

**Table 3:** Allelic carriage and family history of breast cancer

Alleles HLA	Family history of breast cancer		OR (95% CI)	*p* value
	Yes, *N* (%)	No, *N* (%)		
DRB1*11				
Presence	1 (10)	24 (28.57)		Ref
Absence	9 (90)	60 (71.43)	3.6 (0.43–29.97)	0.28
DRB1*12				
Presence	4 (40)	50 (59.52)		Ref
Absence	6 (60)	34 (40.48)	2.2 (0.57–8.4)	0.31
DRB1*11 & 1*12				
DRB1*11+ & 1*12+	1 (10)	15 (17.86)		Ref
DRB1*11− & 1*12−	6 (60)	25 (29.76)	3.6 (0.39–32.87)	0.39
DRB1*11+ & 1*12−	0 (0)	9 (10.71)		NA
DRB1*11− & 1*12+	3 (30)	35 (41.67)	1.28 (0.12–13.38)	0.66

**Table 4:** Allelic carrying and overweight/obesity

Alleles	BMI		OR (95% CI)	*p* value
	Normal, *N* (%)	Overweight/obesity, *N* (%)		
DRB1*11				
DRB1*11+	1 (5.88)	14 (31.11)		Ref
DRB1*11−	16 (94.12)	31 (68.89)	0.13 (0.01–1.14)	0.03
DRB1*12				
DRB1*12+	11 (64.70)	25 (55.56)		Ref
DRB1*12−	6 (35.30)	20 (44.44)	0.8 (0.28–2.22)	0.43
DRB1*11 & 1*12				
DRB1*11+ & 1*12+	1 (5.88)	9 (20.00)		Ref
DRB1*11+ & 1*12−	0 (0)	5 (11.11)	—	NA
DRB1*11− & 1*12+	10 (58.82)	16 (35.56)	0.17	0.10
DRB1*11− & 1*12−	6 (35.30)	15 (33.33)	0.27 (0.02–2.69)	0.37

## 4 Discussion

Once considered a disease of old age, breast cancer now affects much younger women. In our study population, the patients were relatively young (mean age was 45.19 ± 0.9 years). This average age is lower than that found by Bambara et al., 2017, who found average age to be 48.20 ± 12.4 years in their study of a population of Burkinabe women [28]. Since 1997, women with average age in 41–50 years of age group was identified as the most affected by breast cancer in Burkina Faso [29]. In addition, just over a quarter (25.53%) of the patients were under 40 years of age, a higher proportion than the 20.3% found recently in a study by Côte d’Ivoire [30]. In addition, there was a risk of breast cancer in women of age greater than 40 years (OR = 5.01; 95% CI [2.74–9.32]; and *p =* 0.001). This finding seems to correlate with the increase in breast cancer cases, which is generally proportional to the increase in age [31].

The age at the time of diagnosis of the cases ranged from 11 to 57 years. This average age is slightly lower than that found in the study by Côte d’Ivoire, which found 45.21 years as the age of diagnosis [32]. Our results indicate that early breast cancer is increasingly encountered in Burkina Faso. This situation could be related to the youth of the Burkinabe population. Indeed, 77.9% of the Burkinabe population was under 35 years of age in 2019 [33].

In this study, obesity or overweight was not associated with breast cancer risk (OR = 1.4; 95% CI [0.59–3.64]; and *p =* 0.48). In a previous study in Burkina Faso, 18.75% of patients were overweight, which increased the risk of developing cancer in non-multiparous women (*p =* 0.011), but there was no association between obesity and breast cancer [34]. Overall, it is known that African women are plump, so much so that several study cohorts, notably in Nigeria, have not found any risk of cancer linked to obesity [35]. However, postmenopausal obesity contributes to an increased risk of breast cancer in both the Caucasian races [36], black American [37] and black African populations [38].

The treatment of breast cancer depends on several parameters, both economic and technical. In this study, surgery was performed in the majority of patients (92.55% of cases). On the other hand, radiotherapy was used in just over a quarter (26.60%) of cases. These statistics confirm the finding that in sub-Saharan Africa, the treatment of choice was a mastectomy and that radiotherapy is still unavailable to treat breast cancer [39].

In most of these countries, the poor quality of the health systems is associated with the inexistence of a policy of prevention, screening, or management of cancers. Thus, 77% of sub-Saharan women are diagnosed with stage III or IV breast cancer. Hence, the survival rate of breast cancer patients in the country is very low.

The risk of developing breast cancer is proportionally correlated with the family relationship (degree of consanguinity). This risk would also be more accentuated according to the mutated genes found within the family [40]. Our results do not indicate an association between family history and risk of developing breast cancer. However, in Burkina Faso, in 2017, a study found an association between these two factors [28].

The HLA system is one of the most polymorphic gene systems in humans. Our results show that the HLA-DRB1*12 allele was the most frequent (56.63%), while the HLA-DRB1*11 allele was present in 24.49% of the study population. These frequencies are similar to those obtained in a Tunisian population where HLA-DRB1*12 was present in 49% of participants, while HLA-DRB1*11 represented only 14.36% [41]. However, our results are not similar to those of the Allele Frequency Net Database (AFND) accessible via the link http://www.allelefrequencies.net/, which estimates the carriage of the HLA-DRB1*11 allele at 17% and only 1% of the HLA-DRB1*12 allele in a population composed of 53 Mossi (majority ethnic group in Burkina Faso). Another study of 318 Black African descendants from Brazil found that 13.05% expressed the DRB1*11 allele, while 1.72% expressed the DRB1*12 allele [42]. These differences between our results and those of other studies could be related to the number of participants in each study. However, in general, most studies on these two alleles have reported a high carriage frequency of the DRB1*11 allele, while that of the DRB1*12 allele is very low.

Our results show that no risk of breast cancer was associated with the deletion of the HLA-DRB1*11 and DRB1*12 alleles. Various studies have investigated a possible association of genes of the HLA system. HLA class I genes are by far the most investigated. For HLA class II, HLA-DRB and HLA-DQ are the most studied genes. In various populations, two alleles of the HLA gene, namely HLA-DRB1*11 and HLA-DRB1*12, have been associated either with protection against breast carcinogenesis or with a risk of breast cancer [25]. There was no risk of breast cancer associated with carrying or not carrying these two alleles in our study. In addition, research on the associative effect of carrying these two alleles had shown no risk with the family history of breast cancer. Our results are consistent with those found in an Iranian population where the risk of carrying these alleles was not associated with a risk of breast cancer development [43]. The same was the case for studying these alleles in relation to age at the time of diagnosis of breast cancer [44]. In addition, other studies of Arabs in Tunisia [45] and Turkey [46] have not found any risk related to the carriage of HLA-DRB1*11 and HLA-DRB1*12 alleles and breast cancer. On the other hand, another study in Tunisia had found that the allele DRB1*11 was associated with protection against breast cancer in a Caucasian population (*p* = 0.0001). However, they did not find an association between carrying the DRB1*12 allele and a risk of developing this cancer [47]. However, a study in Iran found the contrast of these latest results in Tunisia. Indeed, this study found an association between carrying the DRB1*12 allele and an increased risk of breast cancer (*p =* 0.03). In contrast, the DRB1*11 allele did not show a significant association with this cancer [48]. Another study in a Jordanian population found no association between carrying the HLA DRB1*11 allele and the risk of developing breast cancer [49]. Also, Iran authors found a negative association between the alleles, HLA-DRB1*1301 and HLA-DRB1*0101, and breast cancer, while they found positive associations between the allele HLA-DQA1*0301 and this cancer [44]. Similarly, in Mexico, a Latino population study associated the HLA-DRB1*1602 allele with breast cancer [50]. Recently in Italy, authors have shown that DRB1*11 was rather associated with an increased risk of breast cancer (OR = 2.39 and *p =* 0.0019), while the DRB1*12 allele was not associated with any risk [25]. Also, in Italy, HLA-DRB1*11:01 and HLA-DRB1*10:01 alleles were associated with breast cancer risk [25]. In Turkey, Gun et al. 2012, found no association between HLA-DRB1*03 and breast cancer, while HLA-DRB1*13 was correlated with progesterone receptor expression [46]. In the same study, they found an association between HLA-DRB1*03, HLA-DRB1*13, and protection against this cancer, while HLA-DRB1*04 was linked to a poor prognosis in their study population [46].

In our study, deletion of the HLA-DRB1*11 allele was associated (beneficial effect) with obesity/overweight (OR = 0.13; 95% CI [0.01–1.14]; and *p =* 0.03). Thus, an obese or overweight person with this mutated HLA-DRB1*11 allele has less risk of tumor cell escape from the HLA system. Although obesity is a risk factor for cancer, this result may be related to the protective effect of obesity on cancer occurrence in the black population [35,51].

Thus, these different studies show divergent or even contradictory results depending on the populations investigated, hence the need for more in-depth studies in different populations to establish meta-analyses that can be used for very advanced risk analyses.

This work is a preliminary study of the involvement of HLA gene system alleles in breast cancer in women of Burkina Faso. In addition, we are aware of the statistical bias due to the size of our study population. For this last point, it is useful to emphasize that access to women with breast cancer in Burkina Faso remains somewhat difficult because cancer care is still being improved through universal health insurance and the creation of cancer services in the country’s regions.

## 5 Conclusion

This study explored the frequency of HLA-DRB1*11 and 1*12 alleles and their involvement in breast cancer. Deleting the HLA-DRB1*11 allele is associated with obesity/overweight, which is a risk for cancer development. However, the relatively early age of breast cancer diagnosis requires further investigation of the other HLA gene alleles. In addition, further investigation of a wide range of HLA gene alleles in a large population may provide more information.
